# Supplementary material for: Spatiotemporal coupling and coordinated development of rural revitalization and rural tourism in Jiangsu
Source: PLoS One. 2025 Oct 14;20(10):e0334241. doi: 10.1371/journal.pone.0334241 (PMC12520356; doi:10.1371/journal.pone.0334241)
Supplement: S1 File — (DOCX) [file pone.0334241.s001.docx]

**Link list of paper datasets**

**I. Data sources of Jiangsu Province from 2012 to 2023**

| System | Indicators | Data link URL | Data retrieval mode |
| --- | --- | --- | --- |
| Rural revitalization | Agricultural labor productivity（added value of agriculture, forestry, animal husbandry and fishery/employees of agriculture, forestry, animal husbandry and fishery)） | <https://tj.jiangsu.gov.cn/col/col91733/index.html> | Relevant statistical data from 2013 to 2024 reflect relevant regional economic and social development statistical information of Jiangsu Province from 2012 to 2023. Related data can be found in the "Agriculture" column. |
|  | Gross output value of agriculture, forestry, animal husbandry and fishery |  |  |
|  | Total power of agricultural mechanization |  |  |
|  | Forest coverage rate | <https://tj.jiangsu.gov.cn/col/col87586/index.html> | Data from 2012 to 2022: Statistical Bulletin of National Economic Development of Jiangsu Province from 2012 to 2022 |
|  |  | <https://lyj.jiangsu.gov.cn/art/2019/1/14/art_7197_8002272.html?isappinstalled=0> | Data in 2023: official website of Forestry Bureau of Jiangsu Province |
|  | Ratio of days with good air quality | <https://tj.jiangsu.gov.cn/col/col91733/index.html> | Relevant data can be found in the "Rural Environment" chapter of Jiangsu Statistical Yearbook |
|  | Popularization rate of harmless sanitary toilets | <https://navi.cnki.net/knavi/detail?p=Tkb-n4pqcCGrLSlz97U2n8872CD0CGKIfd0KqkuzJQr34L7jMIyPAA0vbtAuOKdO2Tnc6-7A1-bmnUMgi0FolGZtoWQfILvh6CZ8IyDwO7PahGxREuZv26PP4L33uc-K&uniplatform=NZKPT> | The popularization rate of harmless sanitary toilets from 2012 to 2017 can be found in the "Rural Environment" chapter of the China Environmental Statistics Yearbook (2013-2018). |
|  |  | <https://tj.jiangsu.gov.cn/art/2019/8/26/art_87596_10686615.html> | Data for 2018 |
|  |  | <https://wjw.jiangsu.gov.cn/art/2020/2/26/art_7244_8986325.html> | Data for 2019 |
|  |  | <https://www.zgjssw.gov.cn/fabuting/shengweiwenjian/202110/t20211027_7287408.shtml> | Data for 2020 |
|  |  | / | The relevant data for 2022-2023 cannot be found, and there are missing data. The level of 95% in the above two years is replaced. |
|  | The number of national civilized villages and towns | <http://www.wenming.cn/wmsjk/cjdx_53740/qgwmczmd/> | According to the first to sixth lists, the number of lists in the whole province and each district and city is sorted out. |
|  | The number of intangible cultural heritages at or above the provincial level | <http://www.k.minzunet.cn/mzwhzyk/674771/682705/765186/index.html> | The data includes national and provincial intangible cultural heritages, which are sorted out according to the website data of China Ethnic Culture Resource Bank |
|  | The number of township cultural stations | <https://tj.jiangsu.gov.cn/col/col91733/index.html> | Relevant data can be found in the column of "Culture, Sports and Health" in Jiangsu Statistical Yearbook |
|  | Rural employees（total number of employment-number of employed persons in urban areas） | <https://tj.jiangsu.gov.cn/col/col91733/index.html> | Relevant data can be found in the column "Population, Employment, Wages" in Jiangsu Statistical Yearbook |
|  | The proportion of household expenditure on culture, education, and entertainment | <https://tj.jiangsu.gov.cn/col/col91733/index.html> | Relevant data can be found in the column of "People's Life" in Jiangsu Statistical Yearbook |
|  | Number of people receiving minimum living security |  |  |
|  | Urban-rural residents' income ratio |  |  |
|  | Per capita disposable income of farmers |  |  |
|  | Engel's coefficient of rural residents |  |  |
|  | Per capita floor area of current housing in rural areas |  |  |
| Rural tourism | National Leisure Beautiful Villages | (1) Number of beautiful villages in China before 2021: <https://xccys.moa.gov.cn/xxzlcx/202002/t20200227_6337877.htm?zwkey=afd0fb3c>  (2) List of 2021: <https://www.moa.gov.cn/govpublic/XZQYJ/202111/t20211119_6382538.htm>  (3) List of 2022: <https://www.moa.gov.cn/govpublic/XZQYJ/202211/t20221114_6415375.htm>  (4) List of 2023: <https://www.moa.gov.cn/govpublic/XZQYJ/202309/t20230915_6436612.htm> | The data is collated from relevant website information |
|  | Traditional ancient villages in China | <http://listed.dmctv.cn/index/> | Village data is sorted out according to relevant website information (first batch to sixth batch) |
|  | Leisure agricultural parks and scenic spots of a certain scale | <https://navi.cnki.net/knavi/detail?p=Tkb-n4pqcCESO0wwfBKpnHQhhSdxsyRPoFShd6D3DS3GThWMFWUw6CNW9NTOzvEAwaQpEPPS1YI9DYs8lrRgo_SNwnNPEXfgRD6dURqhQmo=&uniplatform=NZKPT> | The relevant yearbook information of 2013-2024 reflects the statistical information of regional economic and social development of Jiangsu Province from 2012 to 2023.  Before 2021, relevant data of Jiangsu Province can be found from "Leisure Agriculture" in agriculture chapter: scenic spots of leisure agriculture park with certain scale, total number of tourists, tourism income/comprehensive income of leisure agriculture, number of tourism employees/number of leisure agriculture employees; relevant data from 2021 to 2023 can be found in "Rural Industry" in "Rural Revitalization" chapter; |
|  | Total number of tourists |  |  |
|  | Comprehensive revenue of leisure agriculture |  |  |
|  | Number of practitioners in leisure agriculture |  |  |
|  | The proportion of tourism revenue in GDP | <https://tj.jiangsu.gov.cn/col/col91733/index.html> | GDP data can be queried in the column of "National Economic Accounting" in Jiangsu Statistical Yearbook, and then calculated according to the relevant data in the previous section. |
|  | Highway passenger turnover volume | <https://tj.jiangsu.gov.cn/col/col91733/index.html> | Relevant data can be found in the column of "Transportation, Posts and Telecommunications and Service Industry" in Jiangsu Statistical Yearbook |
|  | The number of travel agencies |  | Relevant data can be found in the column "Wholesale and retail, accommodation, catering and tourism" in the Statistical Yearbook. |

**II. Relevant data sources of prefecture-level cities in Jiangsu Province in 2023**

| System | Indicators | Data link URL | Data retrieval mode |
| --- | --- | --- | --- |
| Rural revitalization | Agricultural labor productivity（added value of agriculture, forestry, animal husbandry and fishery/employees of agriculture, forestry, animal husbandry and fishery)） | <https://ceidata.cei.cn>  (accession number: Ceidata3669494647) | Relevant data are obtained through the statistical database of China Economic Network, searched by "index name + prefecture-level city name" in the search column, and the data of 2023 can be selected. |
|  | Gross output value of agriculture, forestry, animal husbandry and fishery | <https://tj.jiangsu.gov.cn/2024/index.htm> | Relevant data can be found in the "Agriculture" column of Jiangsu Statistical Yearbook in 2024. |
|  | Total power of agricultural mechanization |  |  |
|  | Forest coverage rate | <https://lyj.jiangsu.gov.cn/> | The forest coverage rate of each community city in 2023 can be found through the notice announcement of the News Center column on the official website of Jiangsu Province Forestry Bureau |
|  | The ratio of days with good air quality | (1) Nanjing City: <https://www.nanjing.gov.cn/njxx/202401/t20240126_4155696.html>  (2) Wuxi City: <https://bee.wuxi.gov.cn/doc/2024/01/10/4185443.shtml>  (3) Xuzhou City: <https://www.jiangsu.gov.cn/art/2024/5/24/art_88959_11264993.html>  (4) Changzhou City: <https://sthjj.changzhou.gov.cn/html/hbj/2024/HOEBJJFI_0531/25557.html>  (5)Suzhou City: <https://www.suzhou.gov.cn/2024ndzt/mlszgzjz/202406/e9a68ec962e44015befc4de167d5b6e2.shtml>  (6) Nantong City: <http://js.people.com.cn/n2/2024/0605/c360304-40869649.html>  (7)Lianyungang City: <https://www.lyg.gov.cn/zglygzfmhwz/gzdt_msss2024/content/ff800e5a-e895-4bea-bf68-4eca1f8f972b.html>  (8) Huaian City: <http://sthjj.huaian.gov.cn/upload/2024-06/084cf0c7-028d-4e91-b4c3-eb72b004141b.pdf>  (9) Yancheng City: <https://www.yancheng.gov.cn/module/download/downfile.jsp?classid=0&filename=7c3f07723f914a208c6d2b08ebacece8.pdf>  (10) Yangzhou City: <https://sthj.yangzhou.gov.cn/sjzx/ndhjzlgb/art/2024/art_c9e9cae1053f4997b25875063b903a25.html>  (11) Zhenjiang City: <https://www.zhenjiang.gov.cn/zhenjiang/zwyw/202406/6a18c43db8284313a0913be36df96589.shtml>  (12) Taizhou City Jian: <https://hbj.taizhou.gov.cn/hjzl/hjgb/art/2024/art_88f5d460edb0436cbbd8fd9ed249b95f.html>  (13) Suqian City: <https://sthj.suqian.gov.cn/shbj/hjzkgb/202406/b0f3d0eb2bab425891f1aeff19ab7bed.shtml> | Relevant data can be found through the official website news of local governments |
|  | Rural domestic sewage treatment rate | (1) Nanjing City: <https://www.nanjing.gov.cn/hdjl/xwfbh/xwfbhtbnjs2023nsthjzlzkxgqk/>  (2) Wuxi City: <https://sthjt.jiangsu.gov.cn/art/2024/3/11/art_84025_11173520.html>  (3) Xuzhou City: <https://www.jiangsu.gov.cn/art/2024/5/24/art_88959_11264993.html>  (4) Changzhou City: <https://www.changzhou.gov.cn/gi_news/271171325630902>  (5) Suzhou City: <https://www.suzhou.gov.cn/mlxcjs/gzdt/202404/4f01308aea4546e6a3ce63cf1550afbc.shtml>  (6) Nantong City: <https://www.nantong.gov.cn/ntsrmzf/zxta/content/f8bf4041-2d68-436e-831c-ff83a857213e.html>  (7) Lianyungang City: <http://hbj.lyg.gov.cn/lygshbj/zdgz/content/3b1af54b-ce19-4d10-91ae-9c10b5ac8c3c.html>  (8) Huaian City: <http://sthjj.huaian.gov.cn/upload/2024-06/084cf0c7-028d-4e91-b4c3-eb72b004141b.pdf>  (9) Yancheng City: <https://wap.yancheng.gov.cn/vipchat/home/site/1/11140/article.html>  (10) Yangzhou City: <https://sthj.yangzhou.gov.cn/zfxxgk/fdzdgk/xwfbh/art/2024/art_ae400210bb8c4d2bab4587afa46b0efe.html>  (11) Zhenjiang City: <https://www.toutiao.com/article/7376799751695598106/?upstream_biz=doubao&source=m_redirect>  (12) Taizhou City: <https://sk.taizhou.gov.cn/ztlm/tzyj/art/2024/art_8675dc0288e543eeabcf8188ecfe0e27.html>  (13) Suqian City: <https://sthj.suqian.gov.cn/shbj/hjzkgb/202406/b0f3d0eb2bab425891f1aeff19ab7bed.shtml> | It is difficult to find the data on the prevalence rate of harmless sanitary latrines in various districts and cities, so the data on rural domestic sewage treatment rate are used instead here. Relevant data can be found through the official website news of local governments. |
|  | The number of national civilized villages and towns | <http://www.wenming.cn/wmsjk/cjdx_53740/qgwmczmd/> | According to the first to sixth lists, the number of lists in the whole province and each district and city is sorted out. |
|  | The number of intangible cultural heritages at or above the provincial level | <http://www.k.minzunet.cn/mzwhzyk/674771/682705/765186/index.html> | The data includes national and provincial intangible cultural heritages, which are sorted out according to the website data of China Ethnic Culture Resource Bank |
|  | The number of township cultural stations | non-public data | Relevant data are obtained through letters or telephone consultations from the government affairs platform of Jiangsu Province. |
|  | Rural employees（total number of employment-number of employed persons in urban areas） | <https://ceidata.cei.cn>  (accession number: Ceidata3669494647) | Relevant data are obtained through the statistical database of China Economic Network, searched by "index name + prefecture-level city name" in the search column, and the data of 2023 can be selected. |
|  | The proportion of household expenditure on culture, education, and entertainment | <https://tj.jiangsu.gov.cn/2024/index.htm> | Relevant data can be queried or calculated according to relevant data in the column of "Basic Information of Rural Resident Families by Region (2023)" in "Social Economy of Cities and Counties" or "People's Life" in Jiangsu Statistical Yearbook in 2024 |
|  | Number of people receiving minimum living security |  |  |
|  | Urban-rural residents' income ratio |  |  |
|  | Per capita disposable income of farmers |  |  |
|  | Engel's coefficient of rural residents |  |  |
|  | Per capita floor area of current housing in rural areas |  |  |
| Rural tourism | National Leisure Beautiful Villages | (1) Number of beautiful villages in China before 2021: <https://xccys.moa.gov.cn/xxzlcx/202002/t20200227_6337877.htm?zwkey=afd0fb3c>  (2) List of 2021: <https://www.moa.gov.cn/govpublic/XZQYJ/202111/t20211119_6382538.htm>  (3) List of 2022: <https://www.moa.gov.cn/govpublic/XZQYJ/202211/t20221114_6415375.htm>  (4) List of 2023: <https://www.moa.gov.cn/govpublic/XZQYJ/202309/t20230915_6436612.htm> | The data is collated from relevant website information |
|  | Traditional ancient villages in China | <http://listed.dmctv.cn/index/> | Village data is sorted out according to relevant website information (first batch to sixth batch) |
|  | Key villages and towns of rural tourism | <https://navi.cnki.net/knavi/detail?p=Tkb-n4pqcCESO0wwfBKpnHQhhSdxsyRPoFShd6D3DS3GThWMFWUw6CNW9NTOzvEAwaQpEPPS1YI9DYs8lrRgo_SNwnNPEXfgRD6dURqhQmo=&uniplatform=NZKPT> | It is difficult to obtain the number of scenic spots in leisure agricultural parks with certain scale in each district and city, so it is replaced by key villages and towns of rural tourism. Relevant data can be found in the tourism chapter of Jiangsu Yearbook under "Rural Tourism"; |
|  | Total number of tourists | non-public data | Relevant data are mainly obtained through letters or telephone consultations from municipal government platforms at various levels, and belong to non-public data.。 |
|  | Comprehensive revenue of leisure agriculture |  |  |
|  | Number of practitioners in leisure agriculture |  |  |
|  | The proportion of tourism revenue in GDP | <https://ceidata.cei.cn>  (accession number: Ceidata3669494647) | Relevant data are obtained through the statistical database of China Economic Network, searched by "index name + prefecture-level city name" in the search column, and the data of 2023 can be selected. |
|  | Highway passenger turnover volume |  |  |
|  | The number of travel agencies | <https://tj.jiangsu.gov.cn/2024/index.htm> | Relevant data can be found in the column "Wholesale and retail, accommodation, catering and tourism" in the Statistical Yearbook. |
| driving factors for the coupled and coordinated development of rural revitalization and rural tourism | Per capita GDP | <https://ceidata.cei.cn>  (accession number: Ceidata3669494647) | Relevant data are obtained through the statistical database of China Economic Network, searched by "index name + prefecture-level city name" in the search column, and the data of 2023 can be selected. |
|  | General public budget expenditure |  |  |
|  | Urbanization rate |  |  |
|  | The difference in per capita disposable income between urban and rural areas (Urban income-rural income) |  |  |
|  | Per capita consumption expenditure of urban residents |  |  |
|  | Density of the transportation road network（Road route mileage/area） |  |  |
|  | Leisure agriculture business entities | non-public data | Relevant data are mainly obtained through letters or telephone consultations from municipal government platforms at various levels, and belong to non-public data.。 |
|  | Rural tourism resource density | According to the above data, the density of rural tourism resources = the number of rural tourism resources/the area of the region | umber of rural tourism resources = number of leisure and beautiful villages in China + number of traditional ancient villages in China + number of key rural tourism villages and towns |
