# Supplementary material for: Spatiotemporal coupling and coordinated development of rural revitalization and rural tourism in Jiangsu
Source: PLoS One. 2025 Oct 14;20(10):e0334241. doi: 10.1371/journal.pone.0334241 (PMC12520356; doi:10.1371/journal.pone.0334241)
Supplement: Attachment — Submitted filename: Response to Reviewers.docx [file pone.0334241.s002.docx]

Meinqin Ding

School of Air Transport and Engineering, Nanjing University of Aeronautics and Astronautics Jincheng College

Nanjing, Jiangsu, 211156, China

[alading0827@nhjcxy.edu.cn](mailto:alading0827@nhjcxy.edu.cn)

Manuscript Number:PONE-D-25-31895

To: PLOS ONE Editorial Department

Subject: Revised manuscript resubmission(Response to manuscript PONE-D-25-31895)

**Dear Editor ChunguangHu and PLOS ONE Reviewer:**

We appreciate the opportunity to revise our manuscript titled "Spatiotemporal Coupling and Coordinated Development of Rural Revitalization and Rural Tourism in Jiangsu" and are grateful for the insightful comments provided by the reviewers. Those comments are all valuable and very helpful for revising and improving our paper, as well as the important guiding significance to our researches.

We have thoroughly revised the manuscript in accordance with your suggestions. Enclosed with this correspondence are:

1. A manuscript marked with all the changes ("Revised Manuscript with Track Changes") ;

2. A clean version of the revised manuscript("Manuscript");

3. This reply letter("Response to Reviewers").

In the following, we have provided detailed responses to each of the reviewers' comments. Revised portion are marked in red in the paper. Additionally, we have conducted a comprehensive revision of the entire manuscript. In this response letter, the reviewers' comments are presented in italics, and our corresponding changes and additions to the manuscript are highlighted in red text. We have tried our best to make all the revisions clear, and we hope that the revised manuscript meets the requirements for publication.

**Part I: Responding to the Journal's Requirements**

We have ensured that the manuscript fully complies with the formatting requirements of PLOS ONE and have processed all the files as required.

1. *Regarding the copyright of Figure (Figure Number)*

**Response 1:** The map used in the paper is based on the standard map with review number GS (2024) 0650 downloaded from the Standard Map Service website of the Ministry of Natural Resources (http://bzdt.ch.mnr.gov.cn/), with no modifications to the base map. For the proof of the granted permission, please refer to the attached file"Proof of granted permissions".

2. *Regarding the supporting information:*

**Response 2**： We have moved the supplementary figures in the original manuscript to a separate "Supporting Information" file and ensured that each supporting information file has a a legend listed in the manuscript after the references list.

**Part II: Point-by-point Response to the Reviewers' Comments**

**Reviewer #1:***This manuscript presents a comprehensive study on the spatiotemporal coupling and coordinated development of rural tourism and rural revitalization in Jiangsu Province, using panel data from 2012 to 2023. The methods are rigorous, and the results are supported by solid data. The presentation is clear. The manuscript is recommended for publication in PLOS ONE. Some minor revisions are suggested below.*

**Response :** Thank you very much for your strong support of our work, you have provided us with very valuable advice to improve the quality of this paper! We have used your commen to revise the manuscript and have attached a point-by-point response to your comments:

**Comment 1:** *Is there any correlation between driving factors?*

**Response 1:** This is a very good question. We did take into account the possible interactive relationships among the driving factors. We additionally employed the interaction detection function of the geographical detector, focusing on analyzing the effects of the combined action of multiple factors. The findings are presented in Table 5, accompanied by a detailed analysis of their correlations.

According to the interaction analysis results in Table 5, the pairwise interactions of the eight indicators constituting the influencing factors have more obvious effects than single indicators, all showing two-factor enhancement or non-linear enhancement. In particular, the interaction influence between per capita GP and the density of the traffic road network reaches 1, indicating that when the economy develops to a certain level and the traffic conditions are fully improved, the market demand and rural supply can achieve efficient connection. This also confirms the typical characteristic of "efficient flow of urban-rural elements" in Jiangsu as a developed province. Additionally, the interaction effects among per capita GDP and public budget expenditure, public budget expenditure and leisure agriculture businesses, and leisure agriculture businesses and rural tourism resource density all exhibit a q-value exceeding 0.99. When regional economic development reaches a certain threshold, indicated by high per capita GDP, governments possess greater financial capacity to invest in rural tourism infrastructure, ecological governance, and public welfare through budget expenditure. This investment can lower the operational costs for leisure agriculture businesses. Concurrently, abundant rural tourism resources stimulate market participants' investment interest, enhancing the appeal and sustainability of rural tourism and further fostering its integration with rural revitalization.

(See the second paragraph of section "6.2 Analysis of the dynamic mechanism")

**Comment 2:** *Will different models produce the same results?*

**Response 2:** Thank you for highlighting this. To verify the robustness of the results of our main model (coupling coordination degree model), we followed your suggestion and conducted additional sensitivity analyses. The results have been added to Section 5.4.

## **5.4 The robustness test**

The TOPSIS method was applied to reassess the comprehensive development levels of rural revitalization and tourism, yielding Kendall's coefficients of concordance of 0.979 and 0.993, respectively. The significance tests (p < 0.05) confirmed high consistency between the entropy and TOPSIS rankings, demonstrating robustness. Subsequently, a sensitivity analysis was performed, eliminating secondary indicators such as labor productivity, agricultural mechanization power, minimum living guarantee recipients, and cultural expenditure proportions in the rural revitalization system, as well as ancient villages, tourism revenue proportion in GDP, and travel agency numbers in the rural tourism system. The recalculated coupling coordination degree showed negligible change, suggesting minimal impact from indicator selection. Additionally, four cities with varying coordination levels—Xuzhou, Suzhou, Zhenjiang, and Taizhou—were analyzed. The TOPSIS method recalculated their development levels, and the coupling coordination model was reevaluated. The results aligned closely with the benchmark evaluation, confirming the model's robustness.

(See the section "5.4 The robustness test")

**Comment 3:** H*ow is “rural tourism resource density" defined?*

**Response 3:** Thank you for pointing this out. We should define this core indicator more clearly in the paper. We have provided the definitions.

Data for indicators X_1_, X_2_, X_5,_ X_6_, X_7_, and X8 are primarily sourced from city statistical yearbooks and government website. Data for indicator X_3_ are mainly acquired throughthe government information consultations of various cities, while indicator X_4_ data are mainly derived from resources like the Jiangsu Yearbook. The rural tourism resource density is calculated as the average distribution density, defined as the number of rural tourism resources per regional area.

(See the last paragraph of section "6.1 Selection of dynamic factors")

**Comment 4:** *The authors give differentiated policy recommendations: northern Jiangsu should focus on talent attraction and the integration of culture and tourism, while southern Jiangsu should explore mechanisms to facilitate the two-way flow of urban-rural elements. The problem is how could northern Jiangsu attract more talents?*

**Response 4:** Thank you for raising this profound policy issue. We have conducted more in - depth thinking and improvement on it. The specific solutions are detailed in the"7.3 Policy Implications"section .

Jiangsu Province must enhance efforts to cultivate and attract rural talent. The northern region should implement incentive mechanisms to encourage talent return, such as establishing rural tourism entrepreneurship funds and partnering with universities to create training bases. This would motivate college graduates and local talents to return for entrepreneurial pursuits, revitalizing rural areas.

(See the second paragraph of section "7.3 Policy Implications")

**Comment 5:** *Besides data, some surveys, which may reflect some hidden reasons, are highly suggested.*

**Response 5:** We fully agree with your view that qualitative research can better reveal the underlying mechanisms. Since this study is mainly a quantitative analysis based on macro panel data, it is not feasible to incorporate new field surveys in this revision. However,we have proposed it as a future research direction.Your suggestion has greatly helped improve the study's rigor.

Future efforts should integrate business data, such as Alipay's rural consumption records, to refine the model. Concurrently, in-depth interviews can be employed to gather insights into the cognitive disparities between policy implementers and villagers, thereby identifying implicit barriers in policy execution.

(See the section"7.4 Limitations and Future Directions"）

**Comment 6:** T*here are some typos in the manuscript.*

**Response 6:** Thank you for your careful review. We apologize for our carelessness. We have conducted multiple rounds of careful proofreading on the entire manuscript and corrected all the spelling and grammatical errors that were found.

**Reviewer #2:***The manuscript explores the spatiotemporal coupling and coordinated development of rural tourism and rural revitalization in Jiangsu Province, utilizing a combination of entropy-based weighting, coupling coordination degree models, and geographical detectors. This is a timely and policy-relevant topic that adds value to the academic discourse surrounding rural transformation and tourism development, particularly in the context of developed regions in China. The topic is relevant and potentially significant in understanding regional development dynamics, especially in the context of developed regions undergoing rural transformation. The manuscript demonstrates a strong empirical foundation and introduces a critical reflection on classical theories such as resource dependence and industrial integration.*

**Response :** We sincerely appreciate your detailed, pertinent, and highly constructive comments, which have significantly enhanced the depth and breadth of this study. We have used your commen to revise the manuscript and have attached a point-by-point response to your comments:

**Comment 1:***Title and Abstract*

*• What Needs Improvement:*

*The title is overly long and could be more concise without losing clarity. The abstract is informative but densely packed with technical detail (e.g., p-values, q-values) that may not be suitable for a general audience.*

*• Recommendation:*

*Simplify the title while retaining key terms. Revise the abstract to follow a clearer structure (objective, methods, key results, implications), avoid excessive statistics, and highlight the study’s unique contribution.*

**Response 1:** Thank you for your suggestion. We have streamlined the title to emphasize the core content and revised the abstract to enhance its structural clarity by delineating the purpose, methodology, key findings, and conclusion. We have minimized technical intricacies and emphasized the fundamental research contributions.

**The revised title is as follows:**

Spatiotemporal Coupling and Coordinated Development of Rural Revitalization and Rural Tourism in Jiangsu

**The revised abstract now reads:**

Rural tourism is pivotal in addressing the unidirectional urban-to-rural flow of resources, such as labor migration. However, the interaction between rural tourism and rural revitalization in developed regions remains poorly understood. This study establishes an evaluation index system for rural revitalization and rural tourism, examining their interrelationship. Using the entropy method and coupling coordination degree model, we assess the development levels and coordination degrees of these aspects in Jiangsu Province from 2012 to 2023. Furthermore, the geographical detector model is utilized to pinpoint the primary drivers influencing this coordination. The findings are: (1) Both rural revitalization and tourism exhibit significant growth, with southern Jiangsu outperforming the north; (2) The coupling coordination between these systems has strengthened, indicating a profound symbiotic relationship; (3) Spatial distribution differences are notable, with the coupling coordination degree D value in southern Jiangsu being 26.4% higher than in the north. This disparity is primarily attributable to the wider urban-rural income gap and greater fiscal investment in southern Jiangsu. Notably, the traditional "resource dependence theory" appears ineffective in Jiangsu, as the density of rural tourism resources is relatively low. Accordingly, the study proposes differentiated policy recommendations: northern Jiangsu should focus on talent attraction and the integration of culture and tourism, while southern Jiangsu should explore mechanisms to facilitate the two-way flow of urban-rural elements. This research provides a theoretical framework for coordinating "policy-market" dynamics in the rural transformation of developed regions.

(see Abstract）

**Comment 2:** *Introduction*

*• Strength:*

*The introduction provides a relevant overview of urbanization and the need for rural revitalization in China.*

*• What Needs Improvement:*

*Some of the opening statements are too generic. The research gap is not clearly articulated until later in the section.*

*• Recommendation:*

*Clarify the problem statement and theoretical gaps earlier in the introduction. Frame the study’s objective more precisely to distinguish it from past research.*

**Response2 :**Thank you for your feedback. We have significantly revised the introduction section by clearly articulating the research question and theoretical gap at the outset. Additionally, we have provided a more precise delineation of the study's objectives and highlighted its distinctions from prior research.

**The refined content is as follows:**

Amid the growing focus on global urban-rural integration and rural revitalization strategies, rural tourism has emerged as a pivotal component in advancing rural revitalization[1], has attracted great attention from policymakers. Currently, the academic community has widely recognized the interrelationship between rural revitalization and rural tourism, examining the latter's development within the context of the former . However, existing studies mostly rely on interface data, making it difficult to capture dynamic evolution. Notably, the quantitative analysis of high-density ruralization's impact on the coupling mechanism in developed regions remains unexplored. Thus, a thorough investigation into achieving the seamless integration of these elements is urgently required.

With the acceleration of the global urbanization process, a large - scale migration of the rural population to cities has occurred. Villages see labour shortages, recession and social degradation. In most regions, countryside communities are becoming less viable[2].

(See the first paragraph of section "1 Introduction")

**Comment 3:** *Literature Review*

*• Strength:*

*The review covers both domestic and international studies and discusses relevant theoretical perspectives.*

*• What Needs Improvement:*

*The section is overly descriptive and reads more like an annotated bibliography than a synthesis.*

*• Recommendation:*

*Reorganize this section thematically. Focus more on identifying what is not known rather than summarizing what is already established. Include a conceptual framework to clearly justify the study design.*

**Response3 :**Thank you for your expert guidance. The "2. Literature Review" section has been revised to follow a thematic structure, organized as "established consensus→identified research gap→positioning of the current study". A concise overview of the research gap and the positioning of this paper is provided in Section 2.4. Additionally, a schematic diagram has been included to visually represent the theoretical framework and methodology of this study, thereby augmenting the thoroughness of the literature review.

The specific revisions are detailed in the "2. Literature Review" section, marked in red in the revised version. And the a conceptual framework is as follows:

(See the section"2 Literature Review" and Fig 2.）

**Comment 4:***Methodology*

*• Strength:*

*The methods employed (entropy method, coupling coordination degree model, and geographical detector) are appropriate and robust.*

*• What Needs Improvement:*

*The methodological section is lengthy, technical, and lacks interpretive clarity. There is no sensitivity analysis or explanation of why specific indicators were chosen over others.*

*• Recommendation:*

*Condense and clarify the methodological descriptions. Justify the choice of indicators and models with supporting literature. Consider adding robustness checks or model validation.*

**Response4:** Thank you for your valuable feedback and recommendationst. The methodology section has been revised for enhanced clarity and accessibility, with comprehensive literature citations supporting the selection of all indicators. Furthermore, in response to Reviewer #1, Section 5.4 has been included as a robustness check to validate both the model and findings.

The literature citations of relevant indicators are added in the last column of Table 1.

The robustness test is presented in Section 5.4 .

(See Table 1）

## **5.4 The robustness test**

The TOPSIS method was applied to reassess the comprehensive development levels of rural revitalization and tourism, yielding Kendall's coefficients of concordance of 0.979 and 0.993, respectively. The significance tests (p< 0.05) confirmed high consistency between the entropy and TOPSIS rankings, demonstrating robustness. Subsequently, a sensitivity analysis was performed, eliminating secondary indicators such as labor productivity, agricultural mechanization power, minimum living guarantee recipients, and cultural expenditure proportions in the rural revitalization system, as well as ancient villages, tourism revenue proportion in GDP, and travel agency numbers in the rural tourism system. The recalculated coupling coordination degree showed negligible change, suggesting minimal impact from indicator selection. Additionally, four cities with varying coordination levels—Xuzhou, Suzhou, Zhenjiang, and Taizhou—were analyzed. The TOPSIS method recalculated their development levels, and the coupling coordination model was reevaluated. The results aligned closely with the benchmark evaluation, confirming the model's robustness.

(See the section"5.4 The robustness test"）

**Comment 5:** *Results and Discussion*

*• Strength:*

*The results are comprehensive and presented with supporting figures and tables.*

*• What Needs Improvement:*

*The discussion is largely descriptive and does not critically engage with previous literature or explore the implications of the findings in depth.*

*• Recommendation:*

*Enhance the analytical depth of the discussion by comparing results with past studies. Avoid overuse of numerical data in text and focus more on interpretation and significance.*

**Response5:** Thank you for the conmment. We extensively revised the discussion in Section 5 by conducting a thorough comparative analysis of our results with the existing literature. We delved into the underlying reasons for the outcomes, examined the theoretical implications, including potential revisions to the resource dependence theory, and elucidated the practical significance of our findings.

The specific revisions are detailed in the "5. Discussion" section, marked in red in the revised version. The added references are as follows.

*Added references*

Li Y, Long, H., & Liu, Y. Spatio-temporal pattern of China's rural development: A rurality index perspective. Journal of Rural Studies. 2015;38:12-26.

Su MM, Wall, G., Wang, Y., & Jin, M. Livelihood sustainability in a rural tourism destination-Hetu Town, Anhui Province, China. Tourism Management. 2019;71:272-281

Han C. The Ministry of Agriculture actively promotes the development of leisure agriculture and rural tourism. 2017. Available from: <https://www.moa.gov.cn/xw/zwdt/201704/t20170413_5558722.htm>.

Long HL, & Tu, S. Rural restructuring: Theory, approach and research prospect. Acta Geographica Sinica. 2017;4:563-576.

Chen Y, Zhu, L., Du, J., & Hong, W. Evaluation of rural comprehensive development level and obstacle factors in various countries around the world. PLoS One. 2025;20(4):e0317282.

Gertler MS. Rules of the game: The place of institutions in regional economic change. Regional studies. 2010;44(1):1-15.

（See Reference）

**Comment 6:** *Conclusions and Policy Implications*

*• Strength:*

*The conclusions present relevant recommendations for regional development and tourism policy.*

*• What Needs Improvement:*

*The theoretical claims (e.g., revision of resource dependence theory) are ambitious and need stronger empirical validation.*

*• Recommendation:*

*Temper broad theoretical assertions unless backed by comparative evidence. Emphasize practical contributions and highlight potential applications beyond Jiangsu Province.*

**Response6 :**Thank you for your feedback. We will refine the theoretical proposition by narrowing its scope and emphasizing specific policy recommendations derived from the empirical findings of this study. Furthermore, we underscore the practical utility of these recommendations in analogous regions outside Jiangsu Province.

The findings of this study challenge the resource dependence theory. Existing literature has emphasized the central role of resource attractiveness in driving rural tourism[65, 68]. However, the results show that the q-value of rural tourism resource density in Jiangsu Province is only 0.2011, significantly lower than that of general public budget expenditure (X2: q = 0.848) and urbanization rate (X5: q = 0.679). This suggests that in developed regions, policy intervention and urban-rural coordination can partially compensate for the inherent deficiencies in resource endowment, leading to a "constructive ruralization"[1]. This finding challenges the "tourism resource lifecycle theory" proposed by Butler (1980)[54], confirming that policy intervention (e.g., the fiscal proportion in northern Jiangsu exceeding 60%) can overcome resource constraints and achieve "leap-frog development" without relying on endowment, providing a development paradigm for resource-scarce regions.

(See the second paragraph of section"7.1 Theoretical Contribution"）

**Comment 7:** *Data and Reproducibility*

*• What Needs Improvement:*

*Not all data are openly available; some are obtained through government correspondence, limiting reproducibility.*

*• Recommendation:*

*Clearly specify which data are publicly available and which are not. If possible, provide anonymized datasets or aggregated indicators for reproducibility.*

**Response7:** Thank you for your reminder. We will enhance the acquisition instructions to boost reproducibility. We have detailed the origins of each indicator data in section "3.3 Data sources" and "6.1 Selection of dynamic factors"(page21, line474-477). Public data sources encompass the Jiangsu Statistical Yearbook, Jiangsu Yearbook, Statistical Bulletins on National Economic Development (from Jiangsu Province and each prefecture-level city), and official government websites. The total number of tourists, tourism income/leisure agriculture income, number of tourism employees/number of leisure agriculture employees in all districts and cities of Jiangsu Province in 2023 are mainly obtained through letters or telephone consultation from municipal government platforms at all levels, which belong to non-public data. Data cannot be shared publicly. In addition, the link list of the paper's dataset is uploaded as a supporting information file.

**Comment 8:** *Limitations and Future Research*

*• Strength:*

*The authors acknowledge several important limitations.*

*• What Needs Improvement:*

*Some limitations, such as the selection bias in indicators and restricted data scope, could be further elaborated.*

*• Recommendation:*

*Strengthen this section by discussing how future comparative or longitudinal studies (e.g., using county-level data or other provinces) could improve the generalizability of findings.*

**Response 8 :**Thank you for your valuable suggestions for improvement. We will enhance the analysis of limitations and propose ideas for future research expansion. To address data scope limitations, future research could expand to county-level panel data, encompassing 96 county-level units in Jiangsu Province. Additionally, to mitigate indicator selection bias, integrating qualitative research methods like in-depth interviews can enhance the credibility of research findings.

**The refined content is as follows:**

Due to data availability constraints, comprehensive prefecture-level city-scale data for previous years could not be obtained. Furthermore, the limited geographical scope of the prefecture-level city scale (n=13) constrains the statistical power of the geographical detector analysis(p = 0.143 for X_3_). To improve the research's generalizability, future studies could expand to county-level panel data (n=96), measure the evolution patterns over a longer time series, and conduct cross-period dynamic comparisons in combination with the comparison of counties with different development gradients. This approach could enable more accurate identification of the underlying driving mechanisms. When selecting indicator systems, data availability often leads to biases. For instance, opting for "leisure agriculture income" over "rural tourism income" can underestimate the coupling and coordination degree by approximately 12%. Future efforts should integrate business data, such as Alipay's rural consumption records, to refine the model. Concurrently, in-depth interviews can be employed to gather insights into the cognitive disparities between policy implementers and villagers, thereby identifying implicit barriers in policy execution.

(see the section"7.4 Limitations and Future Directions"）

**Reviewer #3:***This study uses the entropy method, coupling coordination degree model, and geographical detector to elucidate the nonlinear dynamics and driving factors of their spatio-temporal evolution in Jiangsu province in China from 2012 to 2023.*

**Response :** We are extremely grateful for the rigorous and professional comments you provided, which have helped us refine numerous details in the manuscript. We have used your commen to revise the manuscript and have attached a point-by-point response to your comments:

**Comment 1:** *The abstract should be reorganized. The contents of main findings should be reduced, and those of methodologies and research contents in this manuscript should be supplemented.*

**Response 1 :**Thanks for the conmment. In accordance with the feedback from Reviewer #2, we have thoroughly revised the abstract to align it more closely with academic conventions by minimizing the inclusion of technical intricacies.

**The revised abstract now reads:**

Rural tourism is pivotal in addressing the unidirectional urban-to-rural flow of resources, such as labor migration. However, the interaction between rural tourism and rural revitalization in developed regions remains poorly understood. This study establishes an evaluation index system for rural revitalization and rural tourism, examining their interrelationship. Using the entropy method and coupling coordination degree model, we assess the development levels and coordination degrees of these aspects in Jiangsu Province from 2012 to 2023. Furthermore, the geographical detector model is utilized to pinpoint the primary drivers influencing this coordination. The findings are: (1) Both rural revitalization and tourism exhibit significant growth, with southern Jiangsu outperforming the north; (2) The coupling coordination between these systems has strengthened, indicating a profound symbiotic relationship; (3) Spatial distribution differences are notable, with the coupling coordination degree D value in southern Jiangsu being 26.4% higher than in the north. This disparity is primarily attributable to the wider urban-rural income gap and greater fiscal investment in southern Jiangsu. Notably, the traditional "resource dependence theory" appears ineffective in Jiangsu, as the density of rural tourism resources is relatively low. Accordingly, the study proposes differentiated policy recommendations: northern Jiangsu should focus on talent attraction and the integration of culture and tourism, while southern Jiangsu should explore mechanisms to facilitate the two-way flow of urban-rural elements. This research provides a theoretical framework for coordinating "policy-market" dynamics in the rural transformation of developed regions.

(See Abstract）

**Comment 2:** *In Line 27 ‘.037 and .156.’, it should add zero before decimal point.*

**Response 2 :** Thanks for your careful checks. We are sorry for our carelessness. The statistical data in the original abstract has been omitted, and various spelling and grammar errors have been rectified.

**Comment 3:** *The international background of the study should be provided in Section Introduction.*

**Response 3:** Thanks for your valuable suggestions. we have incorporated an international perspective at the outset of the introduction, underscoring the significance of this research in examining the Chinese scenario within a global framework.

**The refined content is as follows:**

With the acceleration of the global urbanization process, a large - scale migration of the rural population to cities has occurred. Villages see labour shortages, recession and social degradation. In most regions, countryside communities are becoming less viable[2]. According to the data from the World Bank's 2023 report, the rural population loss rate in developing countries reached 37% from 2000 to 2020. In response, various countries have implemented initiatives aimed countermeasuresat rural revitalization, such as Germany's "Village Renewal" project, Japan's "One Village, One Product Movement and Rural Revitalization Initiative", and South Korea's "New Village Movement"[3, 4]. Different from the private land ownership in Europe, China implements the collective land ownership system and promotes rural construction in an orderly manner through a government - led model.

(See the second paragraph of section "1 Introduction")

**Comment 4:** *The importance of the study should be mentioned in Section Introduction.*

**Response 4:** Thanks for your recommendations. In the introduction, we have underscored the significance of exploring the Chinese case in the global context in this study. Furthermore, we have elucidated the research question and theoretical gap at the outset to underscore the significance of this research.

**The refined content is as follows:**

Amid the growing focus on global urban-rural integration and rural revitalization strategies, rural tourism has emerged as a pivotal component in advancing rural revitalization[1], has attracted great attention from policymakers. Currently, the academic community has widely recognized the interrelationship between rural revitalization and rural tourism, examining the latter's development within the context of the former . However, existing studies mostly rely on interface data, making it difficult to capture dynamic evolution. Notably, the quantitative analysis of high-density ruralization's impact on the coupling mechanism in developed regions remains unexplored. Thus, a thorough investigation into achieving the seamless integration of these elements is urgently required.

With the acceleration of the global urbanization process, a large - scale migration of the rural population to cities has occurred. Villages see labour shortages, recession and social degradation. In most regions, countryside communities are becoming less viable[2]. According to the data from the World Bank's 2023 report, the rural population loss rate in developing countries reached 37% from 2000 to 2020. In response, various countries have implemented initiatives aimed countermeasuresat rural revitalization, such as Germany's "Village Renewal" project, Japan's "One Village, One Product Movement and Rural Revitalization Initiative", and South Korea's "New Village Movement"[3, 4]. **Different from the private land ownership in Europe, China implements the collective land ownership system and promotes rural construction in an orderly manner through a government - led model.**

(See paragraphs 1 and 2 of section "1 Introduction")

**Comment 5:** *The research gap should be identified to show the novelty of this study. Section 2.4 cannot show the research gap in current research progress, the novelty of this study and contribution to the theories.*

**Response 5:**We think this is an excellent suggestion.We have re-written this part according to the Reviewers’ suggestion. we have delineated the deficiencies in current research more explicitly and highlighted the specific gaps that this study seeks to address.

**The refined content is as follows:**

**2.4 Research gaps and the positioning of this paper**

While the relationship between rural revitalization and tourism has been extensively studied, empirical analysis of the long-term, nonlinear dynamics of their coupling and coordination remains limited. Research indicates significant spatial differences in this relationship within Jiangsu, with stronger coupling in the south compared to the north. The specific driving factors and spatial interaction mechanisms contributing to these differences are not well understood, and traditional homogenized models fall short of explaining them. In densely developed regions like Jiangsu, where resource endowments are less prominent, the role of high-level policy interventions and urban-rural synergy in sustaining high coupling and coordination levels is unclear. This raises questions about the applicability of the "resource dependence theory" [54] and highlights a critical gap in both theoretical understanding and practical application. Furthermore, variations in research perspectives have hindered the full development of evaluation indices for existing coupling models. Objectively determining the weights of these indices to accurately reflect their coupling and coordination remains a critical methodological challenge.

Addressing existing research gaps, this paper introduces innovations by analyzing panel data from Jiangsu Province (2012-2023). We develop a multi-dimensional evaluation system, weighted via the entropy method, and employ the coupling coordination degree model to elucidate the dynamic evolution and spatiotemporal characteristics of RR and RT. Utilizing the geographical detector, we investigate the driving mechanisms of spatial heterogeneity. The analysis focuses on testing whether and how "policy intervention" and "urban-rural synergy" can overcome "resource constraints" to foster a high-level coupling coordination pathway, amidst the backdrop of "high-density ruralization" in developed regions. In doing so, the study challenges resource-dependence theory and offers novel theoretical perspectives and differentiated policy foundations for rural transformation in developed regions.

(See the section "2.4 Research gaps and the positioning of this paper")

**Comment 6:** *Why the indicators are selected in Section 3.2 and Table 1? There is no explanation. The evidence or citations should be provided.*

**Response 6:**We sincerely appreciate the valuable comments.We provided literature citations and support for the selection of all indicators in S1Table.

The literature citations of relevant indicators are added in the last column of Table 1.

(See Table 1 ）

**Comment 7:** *Section 3.3 is too simple. The data collection progress, data cleaning and preprocess are missing. Which indicator is from Jiangsu Statistical Yearbook, and which is from Jiangsu Tourism Yearbook, statistical yearbooks of local cities, or relevant government websites? Where individual indicator data were unavailable, historical statistical materials and reports were used to infer the values. How to infer the unavailable data? This section cannot show the reliability of the data, which may affect the results.*

**Response 7:**Thanks for your recommendations. Detailed data sources for the 25 indicators were provided in section 3.3.

**The refined content is as follows:**

**3.3 Data Sources**

This study analyzed 25 evaluation indicators in Jiangsu Province from 2012 to 2023. The data utilized in this study were primarily sourced from the following: Agricultural and farmer-related data, as well as highway passenger turnover volume and travel agency statistics, were obtained from the Statistical Yearbook of Jiangsu Province and the statistical yearbooks of individual cities. Forest coverage rates were gathered from the official website of the Jiangsu Forestry Bureau and the Statistical Bulletin on the National Economic Development of Jiangsu Province. The rate of days with good air quality in each prefecture-level city was collected from local government websites. The popularization rate of harmless sanitary toilets was sourced from the China Environmental Statistical Yearbook and the websites of prefecture-level city governments. The number of national civilized villages and towns was obtained from the official website of the Central Spiritual Civilization Office. Intangible cultural heritage data were retrieved from the China National Cultural Resources Database. National leisure beautiful village data were gathered from the official website of the Ministry of Agriculture and Rural Affairs. Additional data were primarily acquired through information consultation on the Jiangsu Yearbook and the government information consultations of various cities. For missing individual indicator data, interpolation methods were employed to supplement and improve the dataset.

(See the section "3.3 Data Sources")

**Comment 8:** *The standard for the classification of coupling degree C and coupling coordination degree D should be cited.*

**Response 8:**Thanks for the conmment. We supplemented the references for the classification criteria of coupling coordination degree in tabular form in the "4.Research Methods" section.

**The additional Table is shown as follows.**

(see Table 2)

**Comment 9:** *Why the indicators are selected in Table 3? The reasons and explanations should be provided. The data source of these variables should be given.*

**Response 9:**Thanks for the valuable comments.We apologize for the donfusion. We have provided a theoretical rationale for the selection of these driving factors and comprehensively documented the data sources for all factors in section 6.1.

**The refined content is as follows:**

**6.1 Selection of dynamic factors**

Spatial variations in the synergistic development of rural revitalization and tourism are influenced by multiple factors. Drawing on prior research[65, 78], this study analyzes eight indicators spanning economic policy, urban-rural coordination, tourism development, and market dimensions, to analyze the factors shaping the spatial differences in the level of coupled and coordinated development.

(1)Economic policy driving force provide essential support for the integration and coordination of the two systems[62].At this level, per capita GDP (X_1_) and general public budget expenditure (X_2_) are selected as indicators to represent local economic development and government macro-control policies, respectively.

(2)The advancement of tourism plays a crucial role in fostering the synchronized progress of rural revitalization and rural tourism. Essential prerequisites for successful tourism development comprise well-established support service infrastructures and a rich array of tourism resources. In this study, two key indicators, namely the quantity of leisure agriculture operators (X_3_) and the concentration of rural tourism resources (X_4_), are chosen to gauge the hosting capabilities of rural tourism and the appeal of tourism assets.

(3)Urban-rural collaboration can enhance the bidirectional flow of resources, optimize allocation, and foster integrated industrial development, significantly benefiting the synergy between rural revitalization and tourism[79]. This study focuses on two indicators: the urbanization rate (X_5_) and the disparity in per capita disposable income between urban and rural areas (X_6_), to assess regional urbanization levels and living standard differences.

(4)The increase in market size has notably facilitated the migration of tourism, transportation, and other elements from urban to rural areas. In evaluating market dynamics, two metrics, urban per capita consumption expenditure (X_7_) and traffic network density (X_8_), are employed to gauge the consumption capacity and transportation accessibility of the tourism market[80].

Data for indicators X_1_, X_2_, X_5,_ X_6_, X_7_, and X8 are primarily sourced from city statistical yearbooks and government website. Data for indicator X_3_ are mainly acquired throughthe government information consultations of various cities, while indicator X_4_ data are mainly derived from resources like the Jiangsu Yearbook. The rural tourism resource density is calculated as the average distribution density, defined as the number of rural tourism resources per regional area.

(See the section "6.1 Selection of dynamic factors")

**Comment 10:** *The analysis of Section 6.2 should be discussed in more depth combined with policies and development level of the variables.*

**Response 10:**Thanks for the valuable comments.We apologize for the donfusion. Following your recommendation, we have enhanced the discussion and policy implications section by closely examining the disparities in development levels among southern, central, and northern Jiangsu, leading to a more detailed and nuanced analysis of the driving factors.

The specific revisions are detailed in the "6.2 Analysis of the dynamic mechanism" section.

**6.2 Analysis of the dynamic mechanism**

From the single-factor detection results driven by coupling coordination (Table 4), public budget expenditure, leisure agriculture enterprises, urbanization rate, and income gap play a crucial role in this coupling process. In contrast, the impacts of per capita GDP and the density of rural tourism resources are negligible. This contradicts the traditional resource dependence theory (Butler, 1980). The results show that the explanatory power of policy investment (X_2_, q=0.85) exceeds that of resource density (X_4_, q=0.20). This is consistent with the logic of the "de-industrialization transformation" in the Ruhr region of Germany (Gertler, 2010): institutional capital and natural capital can be complementary or collaborative[81].

According to the interaction analysis results in Table 5, the pairwise interactions of the eight indicators constituting the influencing factors have more obvious effects than single indicators, all showing two-factor enhancement or non-linear enhancement. In particular, the interaction influence between per capita GDP and the density of the traffic road network reaches 1, indicating that when the economy develops to a certain level and the traffic conditions are fully improved, the market demand and rural supply can achieve efficient connection. This also confirms the typical characteristic of "efficient flow of urban-rural elements" in Jiangsu as a developed province. Additionally, the interaction effects among per capita GDP and public budget expenditure, public budget expenditure and leisure agriculture businesses, and leisure agriculture businesses and rural tourism resource density all exhibit a q-value exceeding 0.99. When regional economic development reaches a certain threshold, indicated by high per capita GDP, governments possess greater financial capacity to invest in rural tourism infrastructure, ecological governance, and public welfare through budget expenditure. This investment can lower the operational costs for leisure agriculture businesses. Concurrently, abundant rural tourism resources stimulate market participants' investment interest, enhancing the appeal and sustainability of rural tourism and further fostering its integration with rural revitalization.

In summary, the diverse driving forces interact to establish a dynamic mechanism that propels the coordinated development of rural revitalization and rural tourism (Fig 6).

(See the section "6.2 Analysis of the dynamic mechanism")

**Comment 11:** *The clarity of all figures should be improved..*

**Response 11:**Thanks for the comments. All figures have been re-exported in high resolution (300 dpi or higher) TIFF or EPS format using professional software like Origin or ArcGIS and verified by the PACE system.

**Comment 12:** *There are some Chinese parentheses in this manuscript. Please have a check.*

**Response 12:**Thank you for your careful review. We apologize for our carelessness. We have checked the whole text and replaced all Chinese parentheses "（）" with English parentheses "()".

We tried our best to improve the manuscript and made some changes marked in red in sed paper which will not influence the content and framework of the paper. We appreciate for Editors/Reviewers'warm work earnestly, and hopethat the revised manuscript will meet the publication criteria of PLOS ONE.

Once again,thank you very much for your comments and suggestions.

Sincerely,

Meiqin Ding

(Corresponding author)

On behalf of all co-authors
